# A Proposed Novel Neurovascular Mechanism for Brain Network Dysfunction After Traumatic Injury

**DOI:** 10.1007/s10439-026-03999-w

**Published:** 2026-02-26

**Authors:** Taotao Wu, Jared A. Rifkin, Adam C. Rayfield, Keith A. Kroma-Wiley, Dani S. Bassett, David F. Meaney

**Affiliations:** 1 Departmaent of Bioengineering, University of Pennsylvania, Philadelphia, PA United States; 2 School of Chemical, Materials and Biomedical Engineering, University of Georgia, Athens, GA United States; 3 Department of Mechanical and Aerospace Engineering, University of Virginia, Charlottesville, VA United States; 4 Department of Physics & Astronomy, University of Pennsylvania, Philadelphia, PA United States; 5 Departments of Physics & Astronomy, Bioengineering Electrical & Systems Engineering, University of Pennsylvania, Philadelphia, PA United States; 6 Department of Neurosurgery, University of Pennsylvania, Philadelphia, PA United States; 7 240 Skirkanich Hall, 210 S 33rd St, Philadelphia, PA 19104 USA

**Keywords:** Functional connectivity, Neurovascular coupling, Kuramoto model, Finite element model, Neuromodulation

## Abstract

Current research in predicting traumatic brain injury risk focuses on the relationship between head impacts and the likelihood of white matter damage, often overlooking the role of neurovascular coupling in the injury response. To fill this gap, we combined biomechanical and neurodynamic principles to simulate alterations to large-scale resting-state brain activity following head impacts. We simulated cortical neural activity with a network of Kuramoto phase oscillators, using structural connectivity to define coupling and a vascular-informed local resource term to capture neurovascular coupling. By combining the neurodynamic model with a brain mechanics model, we investigated two mechanistic pathways of network dysfunction: (1) white matter damage, reflected in degrading network edges, and (2) local tissue oxygenation decline, reflected in adjusting the resource term at each network node. We simulated 53 real-world head impacts using a vasculature template to evaluate the changes in simulated functional connectivity (FC) and neural dynamics relative to injury outcomes (concussion vs. no concussion). To assess vascular variability, simulations were repeated across 41 individual vasculature maps. Our results show simulated FC changes (measured by Pearson’s correlation) consistently correlated well with injury outcomes, regardless of injury mechanism (AUC = 0.89 and 0.90), However, the two injury models yielded distinct FC patterns as indicated by graph metrics. Vascular variability substantially influenced how injury affected FC, with some brains exhibiting resilience to synchrony disruption depending on their vascular structure. These findings offer testable insight into the neurovascular mechanism of brain dysfunction after TBI and have important implications for individualized protection and treatment.

**Supplementary Information:**

The online version contains supplementary material available at https://doi.org/10.1007/s10439-026-03999-w.

## Introduction

With two million individuals in the USA affected annually [1], traumatic brain injury (TBI) is a serious health concern with high morbidity in the population and significant economic costs in the healthcare system [2]. Caused by the action of external forces on the head, TBI disrupts normal brain function and commonly occurs in vehicle crashes, sports, falls, and assaults. Our understanding of TBI pathophysiology remains incomplete, which hinders the improvement of protective equipment and the development of effective therapeutic interventions. One of the important but understudied components of the pathophysiology of TBI is the perturbation of energy metabolism after TBI. The brain is energy-expensive and consumes around 20% of the body's substrates (primarily oxygen and glucose) through cerebral blood flow (CBF) [3]. The high energy demand of the brain is the product of neural activity [4] and increases in neural activity are associated with transient increases in CBF [5]. The spatial and temporal correspondence between neural activity and CBF formed the basis of functional magnetic resonance imaging (fMRI), a non-invasive, whole-brain imaging technique for mapping and recording interactions among brain areas during specific brain functions. Given the reliance of neurons on energy supply, energy failure after TBI is likely to contribute to neurological dysfunction.

One important factor contributing to energy failure after TBI is the dysregulation of CBF, which could be transient or lasting and has widely been seen in preclinical animal models and human patients [6]. Decreased microvascular CBF was correlated with worse cognitive outcomes after repetitive concussions in mice [7]. Both increased and decreased CBF have been reported after mild TBI (mTBI), a finding that may be influenced by the presence or absence of symptoms and the time between injury and testing [6, 8]. Symptomatic mTBI patients showed a decline in CBF in the absence of structural changes in brain imaging [9]. Acutely after mTBI, decreased regional CBF was found in concussed athletes in arterial spin labeling (ASL) MRI studies [10, 11]. The energy failure after TBI may also be caused by microbleeds [12], microthrombi throughout the vasculature [13, 14], damage to the neurovascular unit (NVU) [15, 16], and blood-brain barrier (BBB) dysfunction [17].

Despite the emerging evidence of energy failure in human and animal TBI [6], the lack of causal mechanisms linking cerebrovascular disturbances to behavioral dysfunction after TBI presents a serious knowledge gap. Although seminal studies in mild TBI demonstrated a clear phase of hypermetabolism followed by a longer phase of hypometabolism in the brain region subject to percussive injury [18], it is less clear how these metabolic changes affect the broader functional connectivity (FC) of the brain in both the acute and post-acute period after injury. Using preclinical animal models to tackle this problem is especially challenging because it is difficult to make precise measurements that link behavior to changes in the connectivity throughout the brain. In comparison, clinical studies can provide measures of both the changes in structural and functional connections in the network after injury and use fMRI to establish time-varying functional connections throughout the brain when subjects perform tasks [19–23]. However, the clinical TBI studies do not provide a direct measure of the mechanical input that caused the TBI, and the fMRI studies do not extend into a large cohort of TBI patients.

Computational models across the time scales – from the moment of impact to the ensuing damage and recovery – can help bridge this critical gap. Already demonstrated as useful for elucidating the mechanisms that lead to dysfunction and predicting disease-induced alterations [24–28], these models can help study potentially new causal factors that can contribute to the early impairment that occurs after a TBI. By using models that connect large-scale neuronal activity across the brain with detailed measures of brain connectivity, these models provide a network-based perspective of cognition. Recently, the network neurodynamic model was coupled with biomechanical finite element (FE) models to merge the mechanical and functional responses of the brain [28]. However, the NVU formed by the vascular network of arteries, arterioles, capillaries, venules, and veins was not considered in these network neurodynamic models. In principle, the supporting vascular network provides the necessary oxygenation to support the local metabolic demands of the tissue. Given that the local metabolic activity is correlated directly with neuronal activity, an emerging feature of neurodynamic models is to represent the local vascular as a resource to influence the amount of neuronal activity that occurs locally. These models are termed resource constraint models because of their potential to constrain or alter neurodynamics [29, 30].

In this study, we aim to elucidate the generative mechanism of how brain function can be affected by the surrounding vascular network by using an appropriately developed resource constraint model. We integrate three primary features into a single computational model of neurodynamics: the explicit connectivity architecture of the brain, the corresponding architecture of the brain vascular network, and the disruption of both the neural and vascular architecture with a head impact. We simulated resting-state neural activity using a system of coupled Kuramoto phase oscillators representing cortical brain regions linked together based on brain imaging data, modifying the oscillator behavior based on resources supplied by the vascular architecture. By including a simulation of the neurovascular unit and its compromise after impact, we find this new model feature could significantly affect the changes in synchronization and flexibility of the network. Moreover, we uncovered two groups of vascular architectures which showed dramatically different responses to impact. This study provides new insight into how altered brain function can result from energy disruption after TBI and the potential heterogeneity in injury susceptibility originating from cerebrovasculature.

## Materials and Methods

### Resource-Constrained Kuramoto Model and Hemodynamic Model

The resting-state neural activity was simulated using the Kuramoto model of coupled oscillators with resource constraints [30], whose coherent behavior obeys the following differential-algebraic delay equations.

1 $$\dot{\theta }_{i} = \mu R_{i} + K\mathop \sum \limits_{j = 1}^{N} C_{ij} \sin \left( {\theta_{j} \left( {t - \tau *D_{ij} } \right) - \theta_{i} \left( t \right)} \right){ }$$

2 $$\dot{R}_{i} = \alpha + D\left( {B_{i} - R_{i} } \right) + \beta {\dot{\theta }}_{{\mathrm{i}}} ; {\mathrm{i}} = 1, \ldots ,{\mathrm{N}}$$

The phase oscillators ($\theta_{i}$) were initialized randomly from a uniform distribution between 0 and 2p. Connections between oscillators were determined using structural network architectures. $C_{ij}$ is the coupling strength between nodes i and j, determined by their respective structural connectivity normalized by the mean non-zero edge weights in the entire network. $D_{ij}$ is the distance matrix determined by the tract length. K defines the global coupling strength, and τ (if exists) defines the global mean delay. The intrinsic frequency of each oscillator ($\mu R_{i}$) depends on a resource level $R_{i}$, and the sensitivity μ of internal velocity to resource level. The parameter α (negative) defines the intrinsic consumption of resource by an oscillator, ${\mathrm{D}}$ defines the diffusion rate of the resource between bath ($B_{i}$) and oscillator, and β (negative) defines the amount of resource consumed per oscillation. We assume the bath level of energy supply for neurons is related to vascular density in the surrounding region, where higher vascular density increases the bath level of energy supply [31].

We tuned the parameters in the model (Table 1) to (i) match the average intrinsic frequency of the oscillators within the range of gamma band (30-80 Hz) as the gamma band power of the local field potential in electrophysiological studies is the band most correlated with the BOLD signal [5] and (ii) achieve moderate synchronization and high metastability, where the model best approximates empirical resting-state functional magnetic resonance imaging [28, 32, 33]. Then, we used Balloon–Windkessel hemodynamic model [34] to simulate blood-oxygen-level-dependent (BOLD) signal by taking $\sin \left( {\theta_{i} } \right)$ as neural activity input, as detailed in the following equations:

3 $$r_{n} \left( t \right) = sin (\theta_{n} \left( t \right))$$

4 $$\frac{{\tau_{0} \partial v_{n} \left( t \right)}}{\partial t} = f_{n} - v_{n}^{{\frac{1}{\alpha }}}$$

5 $$\frac{{\partial f_{n} \left( t \right)}}{\partial t} = s_{n}$$

6 $$\frac{{\partial s_{n} \left( t \right)}}{\partial t} = r_{n} - \kappa_{s} s_{n} - \gamma_{f} \left( {f_{n} - 1} \right)$$

7 $$\frac{{\tau_{0} \partial q_{n} \left( t \right)}}{\partial t} = \frac{{f_{n} \left( {1 - \left( {1 - E_{0} } \right)^{{\frac{1}{{f_{n} }}}} } \right)}}{{E_{0} }} - \frac{{v_{n}^{{\frac{1}{\alpha }}} q_{n} }}{{v_{n} }}$$

8 $$y\left( t \right) = V_{0} \left( {k_{1} \left( {1 - q_{n} } \right) + k_{2} \left( {1 - \frac{{q_{n} }}{{v_{n} }}} \right) + k_{3} \left( {1 - v_{n} } \right)} \right)$$

9 $$k_{1} = 7E_{0}$$

10 $$k_{2} = 2$$

11 $$k_{3} = 2E_{0} - 0.2$$

**Table 1:** Parameters of the resource-constrained Kuramoto model and the Balloon-Windkessel hemodynamic model

Model	Parameters	Description	Value
	K	the global coupling strength	8
	τ	the global mean delay	6 ms
	μ	sensitivity of internal velocity to resource level	10
	$B_{i}$	bath level	$120*\rho {\mathrm{Hz}}$
Resource-constrained Kuramoto model	${\uprho }$	vascular density	Based-on Image
	${\upalpha }$	the intrinsic consumption of resource by an oscillator	− 1
	${\mathrm{D}}$	Diffusion rate of the resource between bath ($B_{i}$) and oscillator,	0.2
	β	the amount of resource consumed per oscillation	− 1
	G	Grubb’s exponent	0.32
	$\kappa_{s}$	Rate of signal decay	0.65 (1/s)
Balloon-Windkessel hemodynamic model	$\gamma_{f}$	Rate of flow-dependent elimination	0.41 (1/s)
	$\tau_{0}$	Hemodynamic transit time	0.98 (s)
	$E_{0}$	The resting oxygen extraction fraction	0.34
	$V_{0}$	Resting blood volume fraction	0.02

The description of the parameters and corresponding values are listed in Table 1. The neurodynamic and hemodynamic simulations were solved using the implicit Runge-Kutta method implemented in the DifferentialEquations.jl package in Julia [35]. Simulations were run for 200 seconds. To remove transient effects, only the last 180 seconds of the simulated BOLD were used. Pearson correlation coefficients between pairs of simulated BOLD signals were calculated, resulting in a symmetric FC matrix for each case.

### Data

#### Injury Data and Diffusion-Weighted Magnetic Resonance Imaging

An existing database of 53 head impacts collected from National Football League games with known head kinematics and injury outcomes was used in this study, 20 of these cases were diagnosed with mild TBI [36, 37]. Apart from the head kinematics and injury outcomes, individual brain information was not available. A generic structural network derived from the human connectome project (HCP) was selected to represent the white matter architecture of these football players. As detailed in Wu et al. (2022), we reconstructed the preprocessed diffusion data [38] using the q-space diffeomorphic reconstruction (QSDR) and conducted whole-brain deterministic fiber tracking to generate whole-brain tractography [39]. We employed a brain atlas [40], empirically determined using fMRI data, to parcellate the cortical brain regions into 100 regions of interest (ROIs). The number of tracts that pass two ROIs was normalized by the total number of tracts to construct the structural connectivity, and the median length of the tracts that connect two ROIs was also calculated to generate the distance matrix. A network that has the closest geodesic distance to the average HCP structural connectivity was chosen as the ‘generic’ structural network.

#### Angiography Data

Cerebral arterial vasculature was obtained from the publicly available time-of-flight angiography (TOF-MRA) database [41]. TOF-MRA data were acquired at a spatial resolution of 0.6 mm using a 3 T Philips Scanner in healthy participants with an average age of 22 (20–31) years old (*n* = 41). The acquisition protocol and processing steps were described in Bernier et al. (2018). The vasculature images were also parcellated to 100 ROIs based on Schaefer et al. (2018). Vascular density in each ROI was computed as a ratio between the voxels identified as a vessel and the total number of voxels per ROI.

### Simulation

**Fig. 1:**
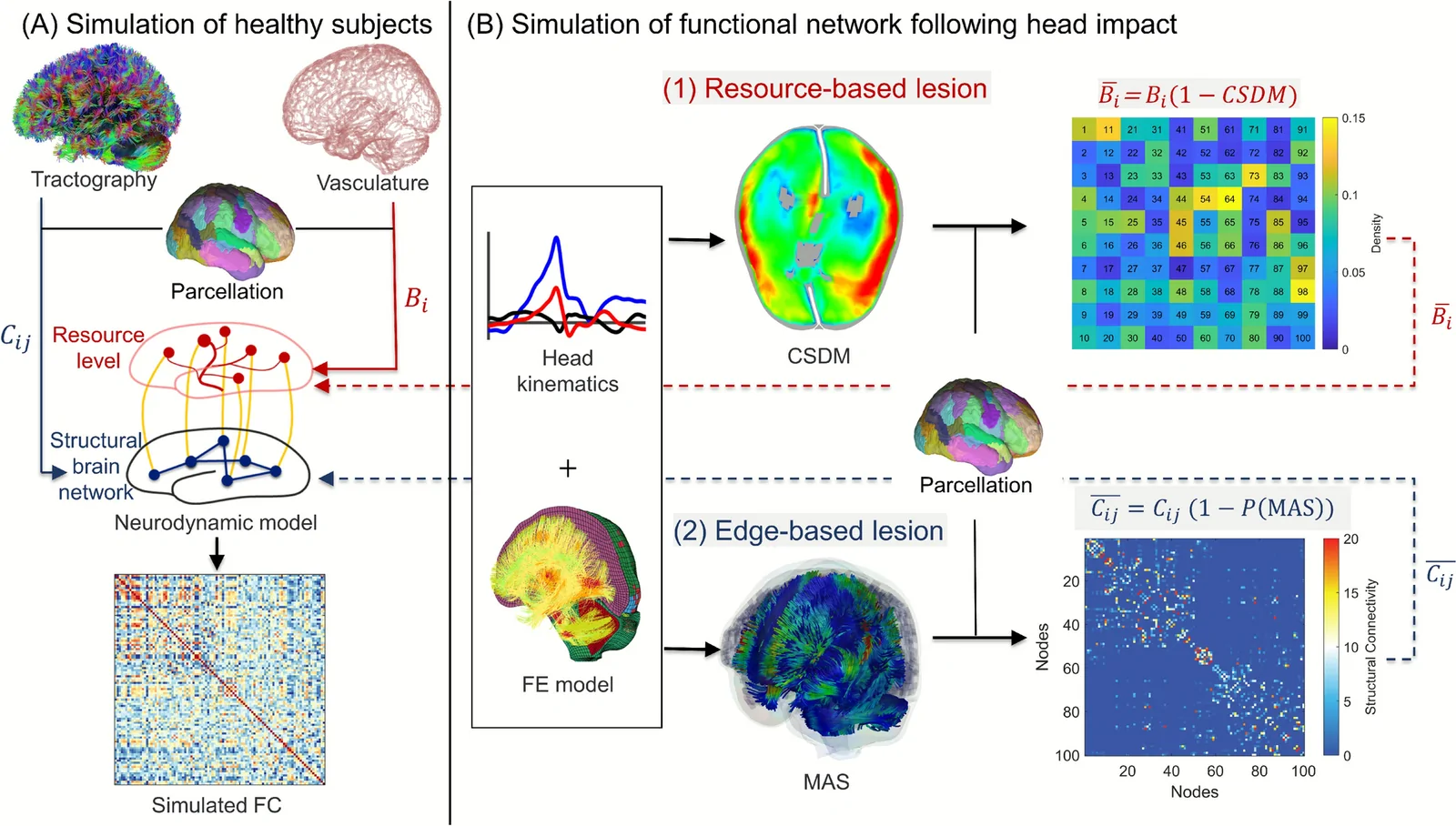
Pipeline for functional brain network modeling. **A** Simulation of the functional network in the healthy subject. **B** Simulation of the functional network following head impact. Two lesion methods were considered: (1) injury to the vasculature led to a decrease in bath level, and (2) damage to the edges (axonal connections) progressively decreased coupling among connected nodes

The overarching pipeline for simulating FC is provided in Fig. 1. Below is the full description of the simulation process.

### Simulation of Healthy Subjects

To simulate the FC of a healthy subject Fig. 1(A), we considered each node (ROI) in the brain network as an oscillator, with coupling strength (*C*_*ij*_) and distance matrix (*D*_*ij*_) determined by the generic structural network derived from diffusion-weighted imaging. The intrinsic frequency for each oscillator is constrained by bath level (*B*_*i*_) that is correlated with the vascular density in each corresponding ROI derived from the angiography data. The vascular density was normalized by the mean vascular density of the 100 ROIs in each subject to maintain a constant overall energy supply in healthy individuals [42].

### Simulation of Head Impacts

#### Finite Element Simulation

To simulate the FC after a head impact, we first used a FE brain model with explicitly modeled axonal tracts [43] to predict the brain deformation induced by the head impact by prescribing the real-world head kinematics using LS-DYNA solver (v971 R9.2.0, double precision; LSTC, Livermore, CA). Full details on the FE brain model and the FE simulation of the 53 football head impacts have been published previously [28]. The element-wise strain (the deformation of brain tissue) resulting from FE simulation was registered to the MRI space. The deformation of each ROI in the Schaefer atlas was represented by the fraction of the region experiencing strain levels higher than a critical threshold of 25%. In the FE brain model, the Schaefer atlas was also used to segment the explicit tracts and compute the tract-wise strain, defined as the maximum tensile strain of the axonal tracts connecting each pair of ROIs.

#### Lesion Method

Our primary lesion method (termed resource-based method) linked the deformation of each ROI to a decline in the corresponding bath level, representing the energy deprivation in each brain region Fig. 1(B). The disrupted bath level for each node after a certain impact can be modified based on the strain metric of each ROI: $\overline{B}_{i} = B_{i} \left( {1 - CSDM} \right)$, where CSDM is the cumulative strain damage measure computed for the brain region. If there was no deformation of the brain during impact (CSDM = 0 in all areas), the bath term was unaffected. If an entire brain region volume exceeded the critical strain threshold (0.25), then the bath term for the region was eliminated.

The second lesion type was based on damage to white matter tracts, where increasing the deformation of white matter tracts led to a reduction of structural connectivity (SC) edge strength. Based on the risk function (*P*) of axonal damage with respect to maximum axonal strain (*MAS*) [44], the modified structural connectome after a certain impact can be modified based on the strain metric in each edge: $\overline{C}_{ij} = C_{ij} \left( {1 - P\left( {MAS} \right)} \right)$. Unlike the edge-based method in our previous work [28], the edge-based lesions in this study lead to the adjustment of intrinsic frequencies of the nodes through the interaction of nodes and redistribution of energy. Subsequent neurodynamic and hemodynamic simulation using the lesioned bath level and/or structural connectome generated the disrupted FC after the impact.

### Data Analysis

**Fig. 2:**
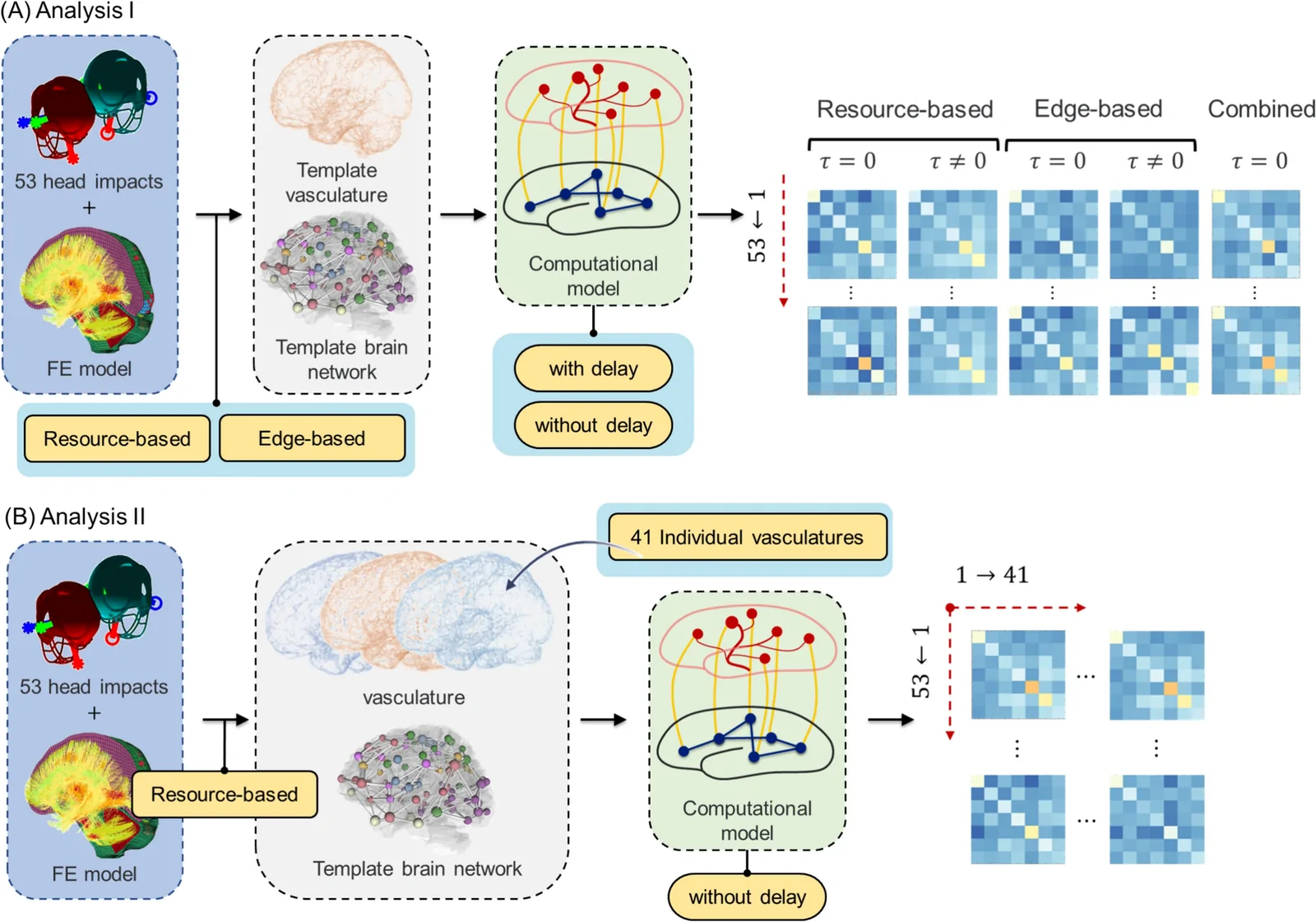
Overview of analyses. **A** Analysis I: Simulation of head impacts using representative vasculature and template brain network with different lesion methods and different delay status. **B** Analysis II: Simulation of head impacts using individual vasculatures and template brain network

Two analyses were performed (Fig. 2). First, using a single representative vascular and structural connectivity architecture which were each an average of the available datasets for structural connectivity [39] and vascular architecture [41]. We investigated two independent lesion methods and the combination of them. We also identified the contribution of signal propagation delay in axons to our results and the combination of lesion methods. For our second analysis, we studied the effect of variability in the vasculature using individual angiography imaging, these simulations were conducted using the 41 individual vasculatures and template network architecture with the resource-based lesion method and without the consideration of signal delay.

For both analyses, we evaluated the correlation between function-related metrics and injury outcomes. The differences between simulated FC of healthy subjects and tested subjects were quantified using the Pearson correlation (PC) of the FC matrices after unrolling into two vectors. We also evaluated the correlation between injury outcome and neural dynamical measures (synchrony, metastability; [28]), and common graph metrics including characteristic path length (CPL) [45], global efficiency (GE) [46], local efficiency (LE) [47], clustering coefficient (CC) [45], and modularity (M) [48]. Negative connections of the FC were removed before the calculation of graph metrics [49].

Synchrony measures the overall coherence of network activity, whereas metastability captures the temporal flexibility of the system by measuring fluctuations in synchrony over time. Graph metrics characterize the spatial organization of information flow by aligning with the canonical principles of global integration and local segregation in brain networks. CPL and GE quantify the ease of global information integration across the network, with shorter paths and higher efficiency indicating faster and more distributed communication between distant regions. LE and CC capture local segregation by measuring how efficiently information can be exchanged within a node’s immediate neighborhood and how densely interconnected those neighborhoods are, respectively. M reflects the degree to which the network partitions into communities of densely connected regions, a feature associated with functional specialization and organized information flow. Together, these metrics provide a compact description of how injury-related perturbations alter the balance between global integration and local segregation in brain networks.

Logistic regression (Equation 14) was employed to correlate binary injury data with continuous metrics (*x*), a and b were the intercept and coefficient of the model, a negative coefficient means the increase of a metric was associated with a reduction in the risk of TBI. The area under the receiver operating characteristic curve (AUC) was used to assess how well the metric discriminates individuals with or without injury, Delong test [50] was used to determine if a function-related metric has statistically worse AUC than the best performer previously found for this dataset [28, 51].

12 $$P_{inj} = \frac{{e^{a + bx} }}{{1 + e^{a + bx} }}$$

## Results

### Impacts Produce a Generalized Loss in Synchronization and Network Flexibility, Regardless of the Injury Mechanism

Broadly speaking, all head impact simulations using a model fitted with the average structural connectome and average vasculature consistently produced a loss in the synchronization and a reduction in metastability (Supp. Fig. A1, A2). When a model utilized only the damage to white matter tracts, the loss in synchronization and metastability were strongly correlated with injury incidence either with or without corresponding time delays (AUC = 0.89/0.89 (synchrony), 0.88/0.86 (metastability)). In comparison, a model simulating the damage to the NVU also showed reasonable correlations to injury incidence either with or without corresponding time delays (AUC = 0.69/0.67 (synchrony), 0.73/0.67 (metastability)), but these predictive values were significantly less than the white matter tract injury model (*p* < 0.01). On average, adding time delays into either injury model only changes the AUC predictive power by 0.7% for the white matter tract model and 2% for the resource-based model. As a result, future modeling efforts only used models without time delays. Combining these two injury mechanisms into a single model (without delays) also led to a strong association between either synchrony or metastability, with predictive performance between either injury mechanism model alone (AUC = 0.83 (synchrony), 0.81(metastability)).

**Fig. 3:**
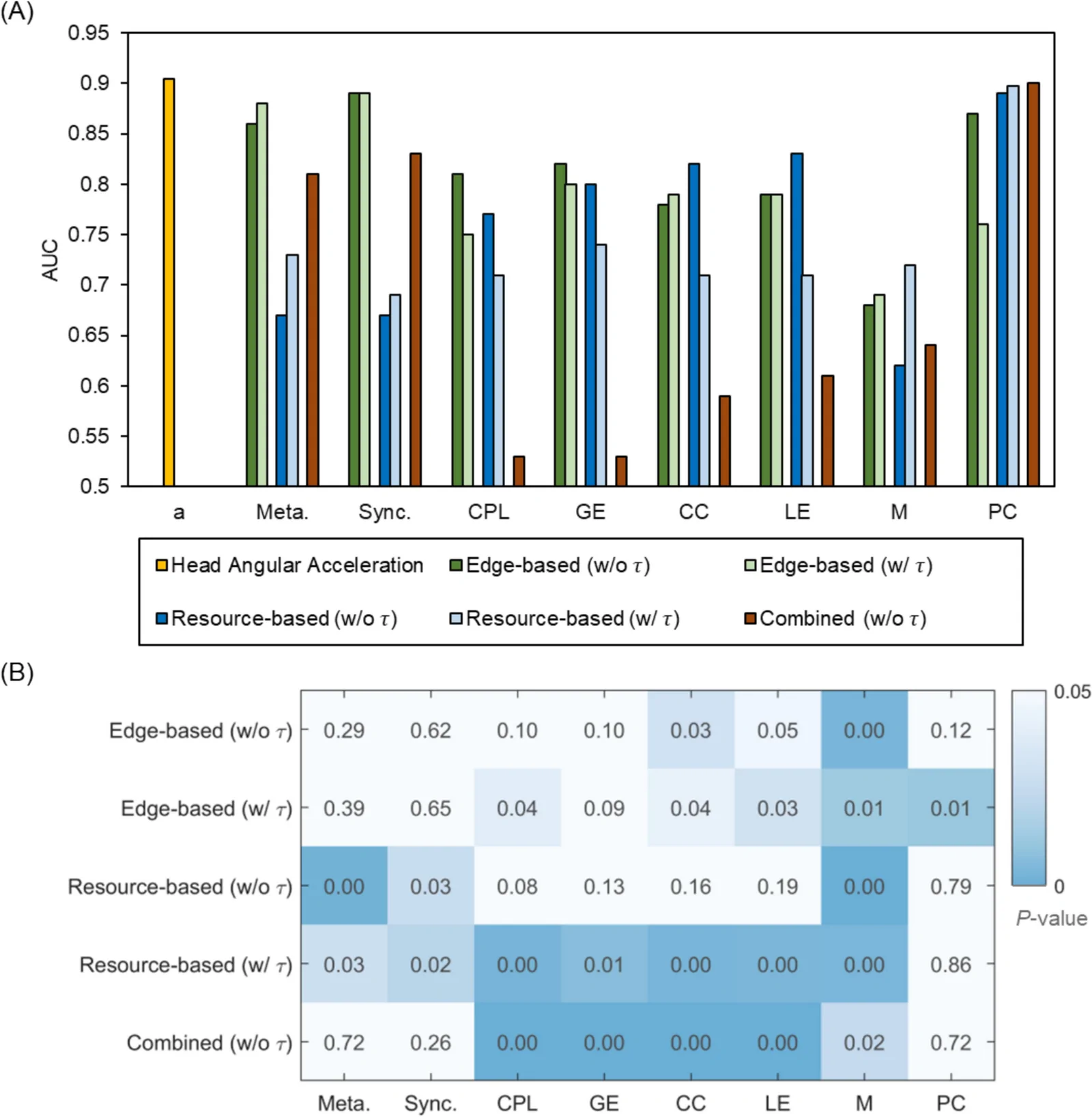
Summary of performance. **A** AUC of metrics. **B** Uncorrected p-values obtained with Delong’s test to determine whether one metric has a significantly different AUC than the measure of the highest AUC (a); Significant values were highlighted with color according to the 5% significant level

Past work also provides measures of the FC network’s ability to transmit information efficiently and quickly, both of which could be compromised after injury. From a large range of possible network-based features to assess information flow, we concentrated on measures of efficiency (characteristic path length (CPL), global efficiency (GE)), transmission (clustering coefficient, CC), and community structure (modularity (M)) in the network (Fig. 3). In comparison to measures of synchrony and metastability, none of these measures performed well for predicting injury occurrence when simulating only white matter tract degeneration (AUC = 0.68–0.82). Alternatively, nearly all of these network measures (except for modularity) offered slight improvement in predicting injury for a model incorporating damage to the vasculature (AUC = 0.77–0.83). Unlike the measures of synchrony and metastability, a combination model that incorporated both the white matter tract and damage to the vasculature generally led to a poorer prediction of injury with all network-based measures (AUC = 0.53−0.64).

All of these measures of either dynamics (synchronization), flexibility (metastability), or an aspect of network structure examined the change in a specific attribute of the neurodynamics. As a final analysis, we used the Pearson’s correlation between the FC matrices prior to, and following, the impact to provide a more generalized measure of the difference between the pre- and post-impact FC states. Regardless of injury mechanisms, the Pearson correlation metrics were a very strong and consistent predictor, which (except for the edge-based lesion with delay) performed as well as, or better than, all previous measures (*p* > 0.05). In comparison, the most successful past predictor of injury probability was based on the peak angular acceleration during impact (a, AUC = 0.90). The injury prediction using Pearson correlation of the FC pre/post impact was not significantly different from peak angular acceleration (a).

**Fig. 4:**
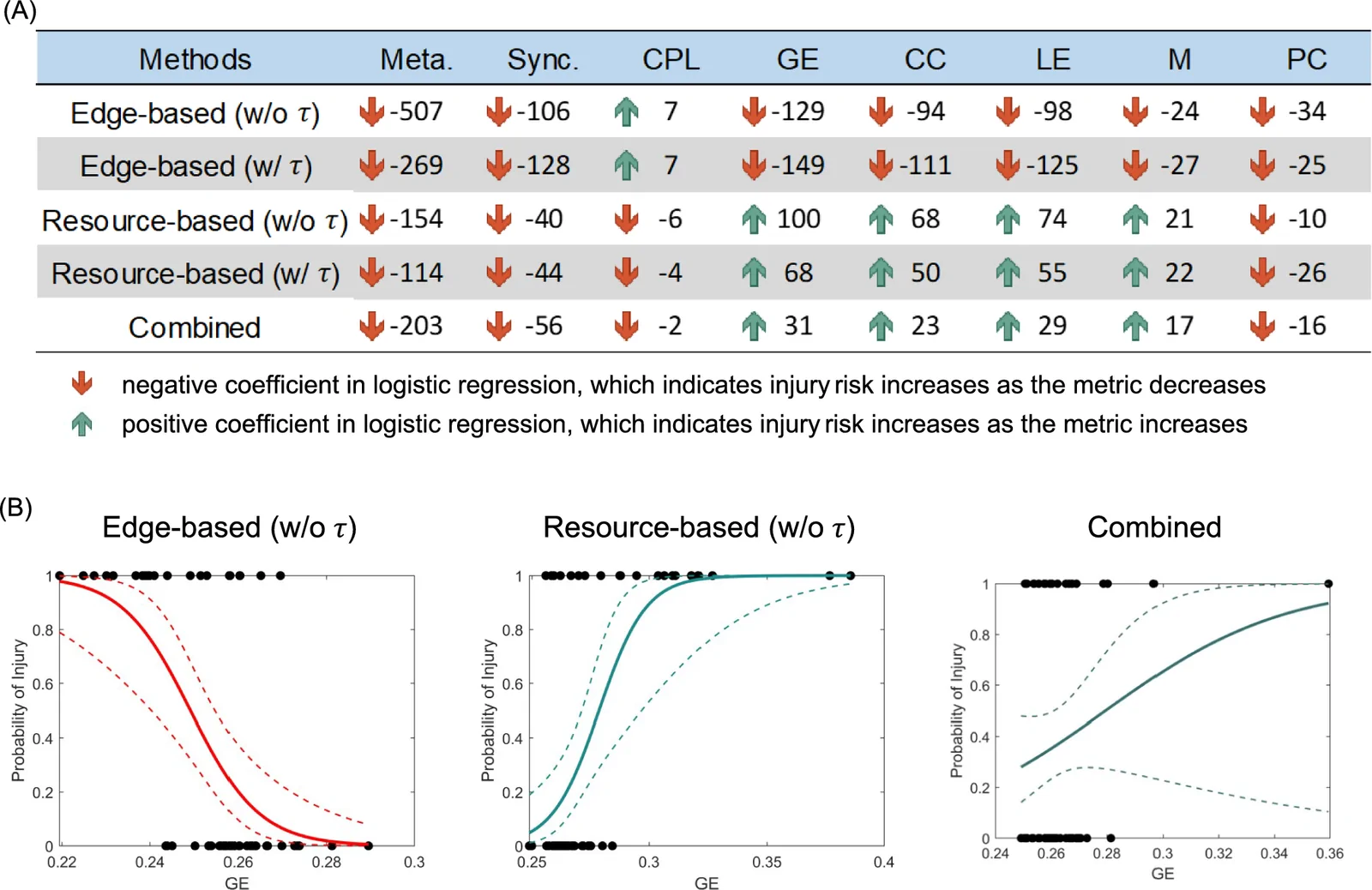
Different injury mechanisms led to different changes in function-related metrics. **A** coefficients in the logistic regression. **B** Logistic regression curves of global efficiency for different injury mechanisms

Despite the similar prediction performance observed between the resource-based and edge-based lesion methods, these results generally showed that resource-based injury led to an increase in the level of global integration (relatively low CPL and high GE) and local segregation (relatively high LE. CC, and M), while both the level of global integration and local segregation decreased as edge-based injury risk increased (Fig. 4).

**Fig. 5:**
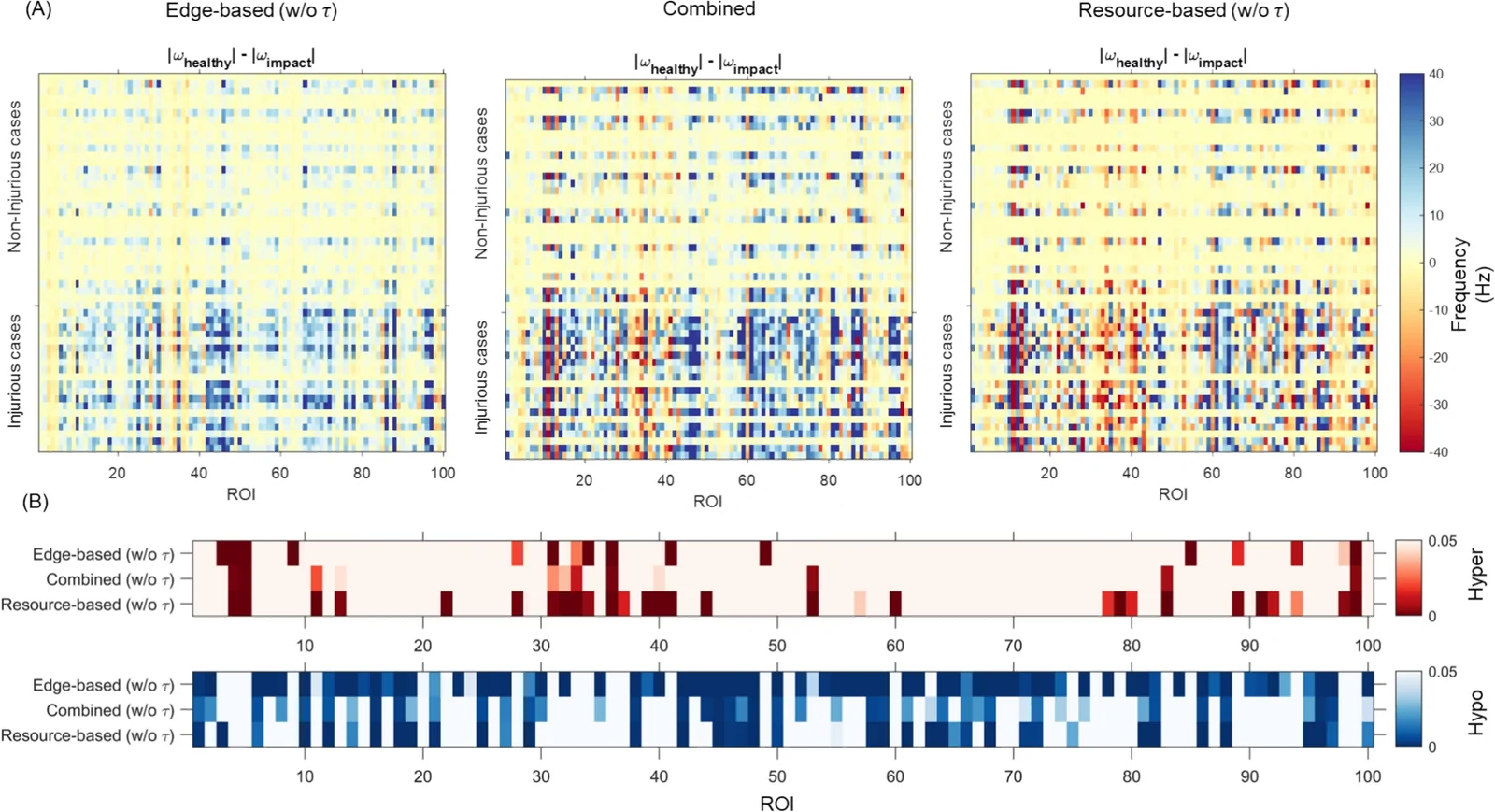
**A** Changes of time-averaged nodal intrinsic frequency after the impacts, positive values indicate decreases of frequencies, negative values indicate increases of frequencies. **B** Uncorrected *p*-values obtained with a one-tailed *t*-test to determine whether the frequencies of injurious cases are significantly higher (hyperexcitation) or significantly lower (hypoexcitation) than those of non-injurious cases for each ROI

Unlike most Kuramoto-based oscillator models, our model adjusts the intrinsic frequency of each node over time. In general, we observed a broad difference in the adjustment of these intrinsic frequencies in either model. Decreasing the strength of white matter connections led to a broad and significant reduction in the average intrinsic frequencies (Supp. Fig. A3). The frequencies for concussion cases significantly reduced comparing to impacts not causing concussion (*p* < < .001). In comparison, damage to the resource-based model caused a mixture of hypoexcitation and hyperexcitation (Fig. 5) which led to smaller differences in the average change in frequencies across all nodes (*p* = 0.002). However, upon comparing the net change in frequencies at each node, a more nuanced picture appeared. For 40 of the 100 nodes, the resource-based model produced a significant reduction in the intrinsic frequency of the nodes, while 29 of the remaining nodes produced a significant increase in the intrinsic frequency. These changes were related to the relative vascular density in this model, as the relative vascular density of hyperexcitation nodes differed from the density of hypoexcitation nodes (*p* = 0.01). In a combined model including both white matter tract and vascular damage, the mixture of hypo- and hyperexcitation persisted (hypoexcitation: 12 of 100 nodes. Hyperexcitation: 46 of 100 nodes). Together, these results a marked change in the predicted nodal dynamics when the model evolved from a traditional, edge-based reduction in network connectivity (dominant hypoexcitation) to a more complete model which accounted for both edge-based and resource-based changes in dynamics (predominant hyperexcitation).

### Role of Individual Vasculature in the Response to Impact

**Fig. 6:**
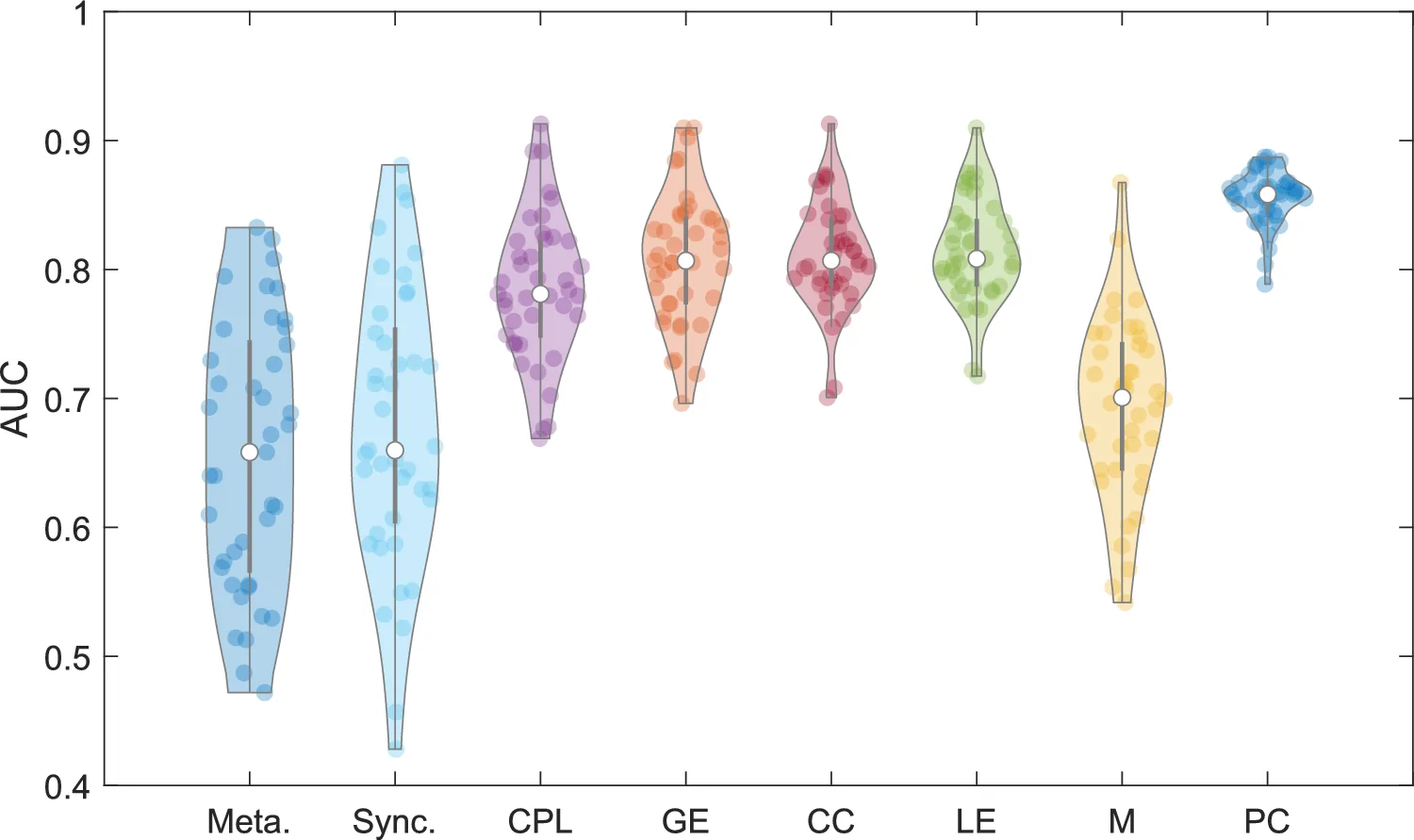
Distribution of AUC for different metrics when using different vasculatures(*n* = 41). In each violin plot, the white dot represents the median and the gray bar in the center represents the interquartile range

**Fig. 7:**
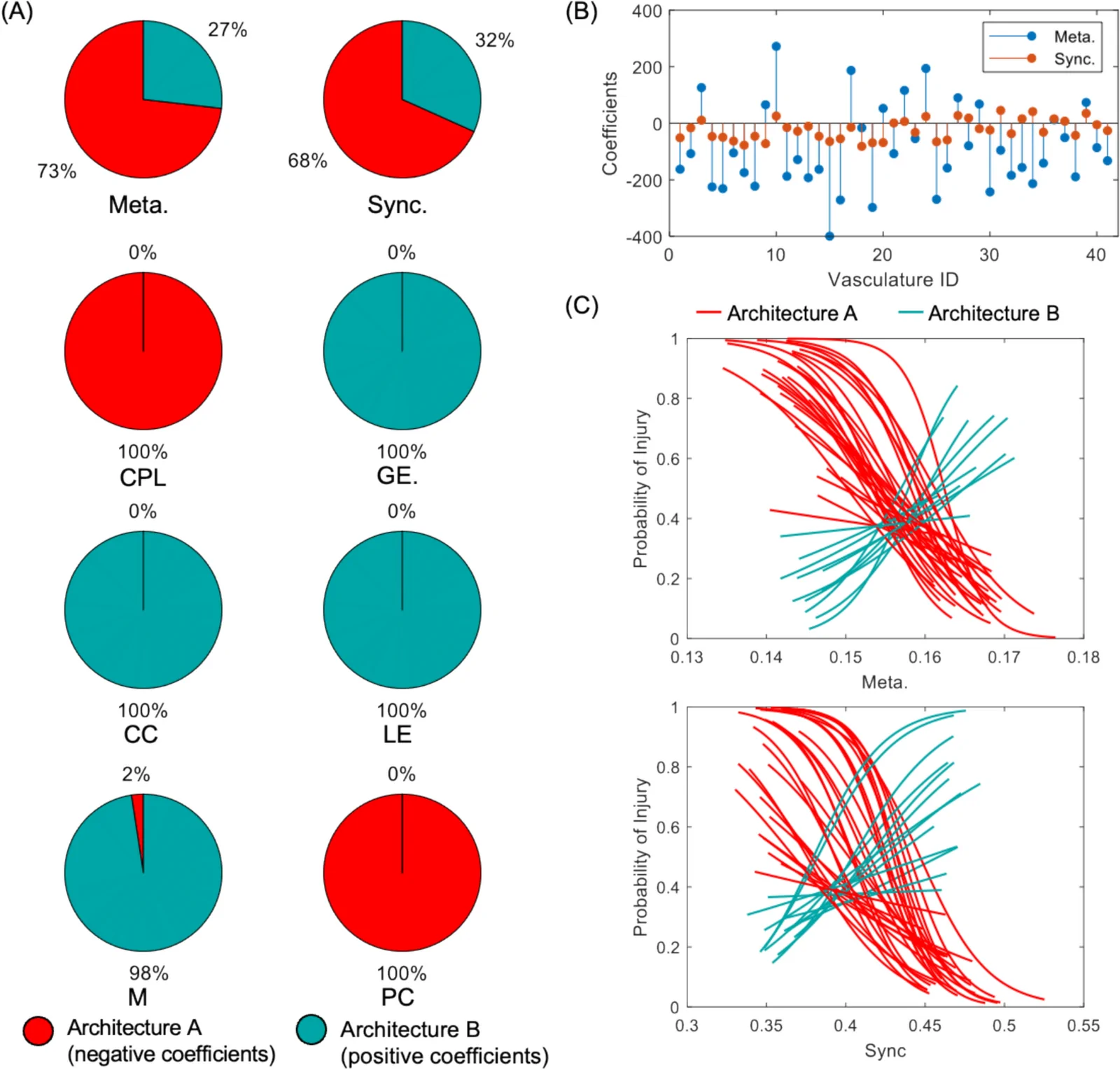
Heterogeneous functional responses caused by different vasculatures(*n* = 41) when using resource-based injury. **A** Distribution of the coefficients in logistic regressions for different metrics when using different vasculature architectures. **B** Coefficients in regression models of metastability and synchrony when using different vasculature architectures, note that the polarities of metastability coefficients correlate well with the polarities of synchrony coefficients. **C** Logistic regression curves of metastability and synchrony when using different vasculatures

Our simulations showed that the explicit consideration of the vasculature, modeled as a bath supply term in the Kuramoto model, could significantly influence the resulting FC among brain areas after impact. We next considered whether the individual variations in vascular architecture would change the predicted neurodynamic response further. To answer this question, we simulated the 53 head impacts using individual vasculatures with resource-based lesions and computed AUC values for each metric (Fig. 6). The performance of PC was least influenced by the variation of vascular architecture, while the performance of synchrony was most influenced. Intriguingly, TBI led to a decline in synchrony for slightly more than two-thirds (28/41) of the vascular architectures but caused an increase in synchronization for the others (Fig. 7). A similar relative fraction of dimorphism was also found in metastability, with 28 showing a decline in metastability with injury and the remaining vascular architectures showing an increase in metastability with injury. The dimorphic response was consistent across synchrony and metastability: 28 of the 28 cases demonstrating an increase in synchrony with injury also showed a decrease in metastability.

**Fig. 8:**
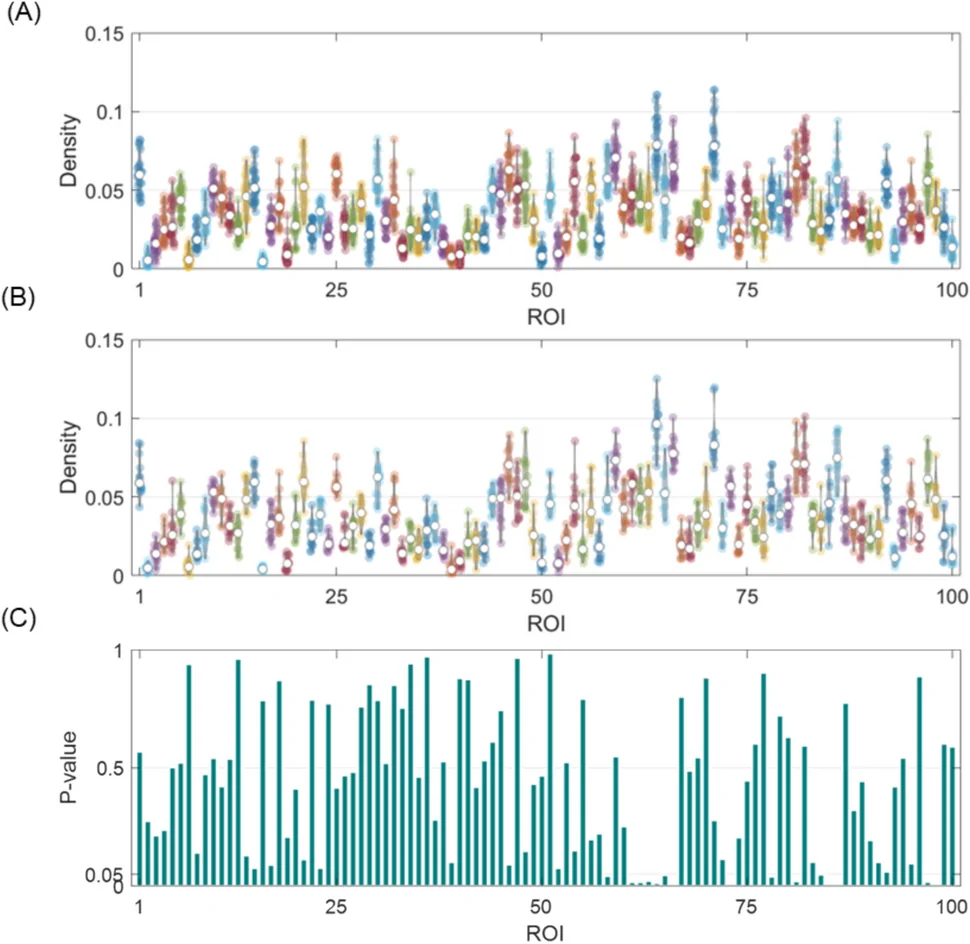
Vascular dimorphism in the relationship between synchronization and injury outcome. **A** Distribution of vascular density in each ROI for vasculature architecture A that led to a negative correlation between synchrony and injury risk (n = 28); the white dot in the violin plot represents the median. **B** Distribution of vascular density in each ROI for vasculature architecture B that led to a positive correlation between synchrony and injury risk (n = 13); the white dot in the violin plot represents the median. **C** *p*-values of each two-tailed test performed for each ROI between the dimorphic vasculature groups. Significantly different ROIs (*p* < 0.05) are listed in Supp. Table A1.

Next, we tested whether there were specific regions of the vasculature that controlled this dimorphic response. We identified 15 regions (Supp. Table A1) that exhibited intergroup differences in vascular density when comparing the vascular maps in models, which showed a decline in synchrony with injury (architecture A) compared to the vascular maps, which showed an increase in synchrony with injury (architecture B) (uncorrected *p* values < 0.05 based on t-tests; Fig. 8). Interestingly, the vascular density in all 15 regions of architecture B were significantly higher than architecture A. We used an average vascular architecture A and progressively increased the vascular densities across the 15 differential groups in a stepwise fashion, eventually achieving a vascular density that matched the average architecture B. As the vascular densities in these 15 brain regions went up by more than 30% from architecture A toward architecture B, the positive correlation between TBI and synchrony gradually transformed into a weak negative correlation (Supp. Fig. A4). In comparison, increasing the density of 15 randomly selected regions did not alter the relationship between TBI and synchrony. Together, these results demonstrate that these 15 regions are key in converting one injury response phenotype (decline in metastability and synchrony with injury) into another phenotype (increase in synchrony and metastability with injury).

Although Architecture B exhibited a positive relationship between injury probability and network measures, this pattern was driven largely by the non-injurious impacts (Supp. Fig. A5A). The 15 regions that distinguish Architecture B have substantially higher vascular density and tend to experience higher strain in these lower-severity impacts (Supp. Fig. A5B). Because the bath level scales with CSDM, regions with a higher initial bath level undergo larger absolute reductions for the same strain damage, producing more pronounced and spatially heterogeneous resource-based disruptions. This heterogeneity reduces local coherence and suppresses global synchrony in the non-injurious regime. For injurious impacts, however, resource-based disruptions become more diffuse across the network, leading both architectures to show comparable synchronization levels. These architecture-specific differences explain why synchrony and metastability increase with injury in Architecture B, despite declining trends in Architecture A.

## Discussion

This work developed an explicitly integrative model to link energy disruption caused by mechanical damage of the cerebrovascular system to brain function disorder and investigated the influence of cerebrovascular architecture variation on susceptibility to brain dysfunction. Although our results show that Pearson correlation between the disrupted and healthy FC consistently correlated well with injury outcomes regardless of injury methods, we found that the two injury methods (resource-based or edge-based) predicted different functional responses after TBI. This discrepancy was not affected by the presence or absence of signal propagation delay. When applying the resource-based injury mechanism to individual vasculatures, we observed that the vascular architecture influenced the changes in neural dynamics and functional networks, resulting in large variations in the correlation between function-related metrics and injury outcome. Finally, we found that these architecture-specific differences in the neural dynamics were focused on a relatively small fraction of nodes (15%) in the vascular network. Together, these results demonstrate a potentially new feature – the energy resources provided by the vascular network – that could play an important role in an *individual’s* response to impact and the subsequent risk of concussion.

This work builds upon our previous study [28], in which we investigated two injury mechanisms—(i) local neurodegeneration leading to decreases in the nodal intrinsic frequency (node-based method) and (ii) damage to axonal connections progressively decreasing coupling among connected nodes (previous edge-based method)—but assumed that the intrinsic frequencies in healthy states only changed by neurodegeneration, and did not consider the additional changes in metabolic demands of injured or recovering tissue. Although dependence on resources may be negligible during a steady state, that dependence becomes important to system behavior when resource-level changes, the connections (coupling strength) in the network change, or when the metabolic demands of the recovering tissue change. The main differences introduced by the consideration of resource constraints were: (i) the nodal intrinsic frequencies were not randomly assigned constants following a distribution, but time-dependent variables shaped by the vascular architecture along with network architecture, (ii) both reducing energy supply (resource-based lesion) and weakening connections (edge-based lesion) alter nodal intrinsic frequencies, and (iii) intrinsic frequencies in some local regions increase after injury due to the reorganization of resources consumption. These characteristics indicate the current work produces a more dynamic change in the network and also provides a more realistic representation of the injury mechanism, as axonal and vascular injuries likely interact with each other to produce a complex pattern of evolving damage [52].

An important distinction emerging from our results is the opposing effect of resource- versus edge-based lesions on network segregation. Because the human brain is organized into spatially compact modules [53], neighboring regions tend to experience similar deformation and similar reductions in metabolic resources. This spatial coherence in vascular impairment causes adjacent nodes to adjust their intrinsic dynamics in parallel, reinforcing locally coherent synchrony and thereby increasing clustering coefficient and local efficiency. In contrast, axonal injury directly weakens the structural connections that normally support tightly interconnected local motifs. The resulting uniform reduction in coupling diminishes the ability of local neighborhoods to maintain coordinated activity, leading to decreases in both segregation and integration.

Our work would potentially provide new insight into some opposing findings in the clinical field. Both increased and decreased FC in local brain networks were reported in TBI patients [22, 54–57] but clinical studies regularly found no statistically significant differences in the global graph metrics of FC (e.g., global efficiency, modularity, clustering coefficient) between TBI patients and healthy control subjects [57, 58]. These general findings of hypo- and hyperconnectivity match predictions from our combined model. As such, these general findings may indicate both cerebrovascular injury and axonal injury coexist in these real-world TBI cases. In a traditional edge-based injury model, we do not see the broad emergence of a mixture of hyper- and hypo-connectivity changes across the network; the changes are predominantly hypo-connectivity. In our combined model, hyperconnectivity occurs when resources become scarce, and because of different strains appearing throughout the brain affect the edge-based coupling among brain regions, some areas are more likely to synchronize and increase their functional connectivity after impact. Some past work provides a basis for our modeling of reduced resources for local dynamics after injury - clinical studies [8, 10, 11, 59] also found both increased and decreased regional perfusion after mTBI. In particular, several ASL studies have identified hyperperfusion in acute and subacute mTBI, including increased CBF in the left striatum and in frontal and occipital regions, as well as elevated venous oxygenation—findings interpreted as blood flow exceeding local metabolic demand in mTBI patients [59]. Increased perfusion after mTBI has also been associated with greater or more persistent symptomatology [8]. These observations demonstrate that mTBI can produce regional increases in perfusion, supporting the plausibility of heterogeneous vascular responses within our resource-based modeling framework. While the hypoperfusion can be easily attributed to the failure of neurovascular coupling, the increases in regional perfusion would also be explained by our model as the intrinsic frequency of certain nodes increases after injury due to the reorganization of the whole system. Since blood circulation is a relatively closed system, blood flow may be diverted to injured sites in response to a recent decrease in flow, such a ‘compensation and regulation mechanism’ is consistent with the observation of cerebral cellular metabolism in visual perception [60].

Due to the close entanglement between neural network and vasculature, we expected variability across vasculature to influence susceptibility to TBI. The angiography dataset represents a tight demographic profile of healthy adults between the ages of 20 and 31. Given this limited age range, we have likely underestimated the variations of cerebrovascular architectures in the general population. Reduced visibility of vascularity of distal arteries in the intracranial vasculature has been found in older adults (56-85 years old) [61], implying that the general resources available may decline with age. Even in the tight demographic group we studied, the variation of vasculature in certain brain regions underpins the changes in the synchronization behavior of the brain after TBI. Impaired network synchronization has been found in TBI [62] and various other cognitive disorders [63]. The discrepancy in synchrony responses indicates individualized injury susceptibility: individuals with higher vascularity in these certain regions are more resilient to TBI than others. It is worth noting that the most significant differences in vascular density appeared in nodes located within the right hemisphere, distributed across 6 of the 7 subnetworks categorized for the human brain. The impact conditions studied are complex and multidirectional. For this reason, we could not determine whether this asymmetry in vascular density would confer different injury risks if the impacts were applied in different directions. It is well known that injury risk is influenced by the direction of impact [64, 65]. However, the path of impact further affects the exact trajectory along which the force travels, such as from anterior to posterior (front to back) or the reverse. This distinction is crucial because the brain's structure, including its vasculature, is not symmetrical; various regions may have different densities of blood vessels and other tissues that influence how forces are distributed and absorbed.

One critical assumption of this study is that the energy supply for neurons is constrained by vascular density. The latest theories consider that the brain at rest is already consuming energy at the limits of blood supply [31] because of the small variations in CBF throughout the day in humans [42] and the linear dependence between blood flow at rest and local capillary density in animal models [66]. Given the relatively homogenous capillary density compared to neuronal density in the brain, regional rates of energy use might be mostly constrained by capillary density [31, 67]. Unfortunately, our source of arterial images has a limited spatial resolution of 0.6 mm. Future work would benefit from angiography data with a higher resolution if available. Nonetheless, prior studies show that regional vascular densities measured from large vessels correlate well with both venous maps [41] and higher-resolution datasets (0.3 mm) [68], suggesting that macrovascular density captures the dominant regional gradients relevant to our resource term. Higher-resolution microvascular data would likely increase inter-subject variability rather than fundamentally change the node-level constraints, potentially sharpening predictions of individual susceptibility. Apart from the vascular density, another limitation is that the resource-constrained Kuramoto model depends on several poorly known parameters. For example, the amount of intrinsic consumption (α, maintaining the neurons and glial cells themselves) and the amount of energy consumption for oscillation ($\beta {\dot{\theta }}_{{\mathrm{i}}}$, synaptic signaling) are difficult to ascertain. Our model was tuned to ensure that the bulk of the energy supply is estimated to be consumed at the synapse [4]. A sensitivity analysis of these parameters (α, β, μ, and D) revealed that the model outcomes were not significantly dependent on these parameters within physiological ranges, where the parameter values produced desirable mean intrinsic frequency, synchrony, and metastability. Some parameters can be absorbed to reduce our system to a minimal number of unique parameters [30]. Since the current parameters are biologically relatable, we kept the current form in the hope of future refinement when relevant data are available. Given the energy supply of the brain in the undisturbed state is already constrained by the capillary supply of blood and oxygen to the tissue, either disruption to the overall CBF after TBI or redistribution of CBF caused by local impairment is expected to influence normal brain function. Blood circulation in the brain can be approximated as a closed system, another possible improvement to this study is to model the neural and vascular networks in a multilayer network manner [69], which allows individual sources to interact with each other and individual oscillators to compete for a common source.

Our findings indicate that changes to resource supply and white matter integrity both play a vital role in the network synchrony disruption and cognitive dysfunction after TBI. The frequency adaptation phenomenon resembles the shifts in electroencephalography (EEG) frequency components that occur after TBI. The EEG of TBI patients most often shows decreased alpha power [70, 71], but increased delta power [70, 72, 73]. Although we tuned the neurodynamic model to gamma-band activity due to its strong correspondence with spontaneous BOLD fluctuations, the framework is flexible and can be extended to lower-frequency regimes (e.g., alpha or beta) that are more relevant to EEG-based assessments, a direction that will be valuable for future multimodal validation. It also provides a new perspective for a conceptually similar technique - clinical neuromodulation - which stimulates certain regions to nudge the brain network to different synchronization patterns [74] and improve cognitive functions [63]. Many have considered neuromodulation a promising tool for the treatment of TBI [75–77]. Our findings indicate that simultaneous changes in neuronal connectivity and local tissue perfusion intrinsic ‘injury modulation’ mechanism, which could be key to an important recovery mechanism that is currently not well known. If correct, this may reveal natural recovery pathways that the brain can activate in concert with clinical neuromodulation. Alternatively, our model may offer additional insight into how neuromodulation may work differently in the traumatically injured brain.

In summary, we proposed a computational model that probes the role of brain energy supply constraints in TBI. Our results show that energy failure and white matter integrity disruption differentially change FC patterns, although the disruption of FC consistently correlated well with injury outcomes regardless of injury mechanisms. Cerebrovascular variability introduced a large variation in the performance of correlating injury with measures from FC. Higher vascularity in certain brains mitigated the negative impact of TBI on synchronization, highlighting a potential source of heterogeneous injury susceptibility within the population. These findings suggest that vasculature differences could inform individualized protection strategies by identifying populations or brain regions at greater risk from specific impacts. For example, regions with lower vascular density, which are more vulnerable to synchronization impairments, might benefit from enhanced protective measures, such as targeted helmet designs or improved safety protocols. Overall, the present findings have fundamental implications for both individualized protection and recovery, providing new diagnostic insights into functional impairments after TBI, leading to clear, testable predictions, and opening new lines of investigation into the neurovascular mechanisms underlying neurotrauma.

## Supplementary Information

Below is the link to the electronic supplementary material.Supplementary file1 (DOCX 6039kb)

## Data Availability

The diffusion MRI and functional MRI data are available through Human Connectome Project (https://www.humanconnectome.org/study/hcp-young-adult). The TOF data is publicly available on Github (https://github.com/braincharter/vasculature). The football impact data is available through Biokinetics and Associates Ltd.
