# Supplementary material for: A Proposed Novel Neurovascular Mechanism for Brain Network Dysfunction After Traumatic Injury
Source: Ann Biomed Eng. 2026 Feb 26;54(9):3175–91. doi: 10.1007/s10439-026-03999-w (PMC13522068; doi:10.1007/s10439-026-03999-w)
Supplement: Supplementary file 1 [file 10439_2026_3999_MOESM1_ESM.docx]

**SUPPORTING INFORMATION**

***Section A1. Supplementary Results of Analysis I***

*Figure A1: Visualization of simulated FCs for an injurious and non-injurious impact.*

*Figure A2: Visualization of the average FCs for the injurious and non-injurious impacts.*

*Figure A3:* Comparison of intrinsic frequencies across all regions for injurious and non-injurious impacts. Statistical significance is indicated by stars: * for p ≤ 0.05, ** for p ≤ 0.01, and *** for p ≤ 0.001.

***Section A2. Sensitivity Analysis of Vascular Dimorphism***

*Figure A4: Effects of regional vascular variations on synchronization responses after head impacts. (A) Increased vascular density (normalized) at the 15 significant regions; (B) Increased vascular density (normalized) at 15 random regions; (C) Changes of the correlation between synchrony and injury outcome as the vascular densities in the 15 significant regions increase gradually; (C) the correlation between synchrony and injury outcome as the vascular densities increase by 50% at 15 random regions, results showing 5 different random seeds of 15 regions.*

*Figure A5: Differences in synchronization between vascular architectures and distribution of tissue deformation within subjects. (A) Distribution of global synchrony for Architecture A and Architecture B across all impacts. Architecture B exhibits lower synchrony than Architecture A in the non-injurious regime; (B) Subject-wise visualization of the spatial distribution of tissue strain, with the 15 regions that differ in vascular density between Architecture A and B highlighted in blue.* *Subjects 1–33 correspond to non-injurious impacts, and subjects 34–53 correspond to injurious impacts.*

*Table A1: Significant regions in Schaefer atlas that causes vascular dimorphism in synchronization*

| Subnetwork | Name | ID |
| --- | --- | --- |
| Visual | 7Networks_RH_Vis_8 | 58 |
| Somatomotor | 7Networks_RH_SomMot_3  7Networks_RH_SomMot_4  7Networks_RH_SomMot_5  7Networks_RH_SomMot_6  7Networks_RH_SomMot_7  7Networks_RH_SomMot_8 | 61-66 |
| Dorsal Attention | 7Networks_RH_DorsAttn_FEF_1 | 73 |
| Ventral Attention | 7Networks_RH_SalVentAttn_Med_2 | 78 |
| Frontoparietal | 7Networks_RH_Cont_Par_1  7Networks_RH_Cont_PFCl_2  7Networks_RH_Cont_PFCl_3  7Networks_RH_Cont_PFCl_4 | 81-86 |
| Default | 7Networks_RH_Default_PFCdPFCm_2  7Networks_RH_Default_PFCdPFCm_3 | 97-98 |
